# Real-time colour hologram generation based on ray-sampling plane with multi-GPU acceleration

**DOI:** 10.1038/s41598-018-19361-7

**Published:** 2018-01-24

**Authors:** Hirochika Sato, Takashi Kakue, Yasuyuki Ichihashi, Yutaka Endo, Koki Wakunami, Ryutaro Oi, Kenji Yamamoto, Hirotaka Nakayama, Tomoyoshi Shimobaba, Tomoyoshi Ito

**Affiliations:** 1 Graduate School of Engineering, Chiba University, 1-33 Yayoi-cho, Inage-ku, Chiba, 263-8522 Japan; 2 Applied Electromagnetic Research Institute, National Institute of Information and Communications Technology, 4-2-1, Nukuikita-machi, Koganei, Tokyo, 184-8795 Japan; 3 Institute of Science and Engineering, Kanazawa University, Kakuma, Kanazawa, 920-1192 Japan; 4 Center for Computational Astrophysics, National Astronomical Observatory of Japan, 2-21-1 Osawa, Mitaka, Tokyo, 181-8588 Japan

## Abstract

Although electro-holography can reconstruct three-dimensional (3D) motion pictures, its computational cost is too heavy to allow for real-time reconstruction of 3D motion pictures. This study explores accelerating colour hologram generation using light-ray information on a ray-sampling (RS) plane with a graphics processing unit (GPU) to realise a real-time holographic display system. We refer to an image corresponding to light-ray information as an RS image. Colour holograms were generated from three RS images with resolutions of 2,048 × 2,048; 3,072 × 3,072 and 4,096 × 4,096 pixels. The computational results indicate that the generation of the colour holograms using multiple GPUs (NVIDIA Geforce GTX 1080) was approximately 300–500 times faster than those generated using a central processing unit. In addition, the results demonstrate that 3D motion pictures were successfully reconstructed from RS images of 3,072 × 3,072 pixels at approximately 15 frames per second using an electro-holographic reconstruction system in which colour holograms were generated from RS images in real time.

## Introduction

Electro-holography is a technique that can reconstruct three-dimensional (3D) motion pictures by switching holograms using a spatial light modulator (SLM)^1–12^. This technique uses a computer-generated hologram (CGH), and several methods are available for hologram generation. The point-based method generates holograms from a 3D object based on point cloud data^13–18^. Although this method involves simple calculations, the computation time typically depends on the number of point clouds and the number of pixels in a hologram. This indicates that it is significantly difficult to realise real-time hologram generation when large holograms are generated from complex 3D objects. In addition, hidden surface removal and gloss reproduction processes are required in hologram generation^19–21^, which results in increased computation time. Conversely, previous studies have proposed a hologram generation method based on light-ray reproduction^22,23^. This method can realise hidden surface removal and gloss reproduction by rendering computer graphics (CG) in a pre-process. However, the rendering and hologram planes are in the same position, and the rendered image becomes blurred when the object is distant from the hologram plane. Subsequently, the ray-sampling (RS) plane method has been proposed^24^. The RS plane method suppresses blurring of the rendered image by rendering on the RS plane set up near the object. The computational cost of this method is lower than that of other point-based methods because it employs fast Fourier transform (FFT). However, the computational cost is still too high for real-time reconstruction of 3D motion pictures. Thus, we focus on the computation time of the aforementioned method and specifically aim at speeding up colour hologram generation from 3D polygon data based on an RS plane using a graphic processing unit (GPU) to reconstruct 3D motion pictures in real time. In addition, GPU memory buffer overflows is one of the technical difficulties in implementing the RS plane method onto a GPU. We also aim to reduce the amount of GPU memory used in order to achieve high-quality 3D images.

## Results

We used the following environment to generate a hologram: Microsoft Windows 10 Enterprise; 3.40-GHz Intel Core i7-6800K central processing unit (CPU) (full use of six cores) with 32 GB memory; Microsoft Visual C++ 2013; NVIDIA Geforce GTX 1080 GPUs (1,823-MHz GPU clock, 5,005-MHz memory clock, 8,192 MB memory and 2,560 cores) and compute unified device architecture (CUDA) version 8.0 as an integrated development environment for GPU. For the reconstruction environment, we set the hologram resolution to 1,920 × 1,080 pixels, the pixel pitch to 8.0 μm, the blue wavelength to 450 nm, the green wavelength to 532 nm, the red wavelength to 650 nm and the propagation distance to 0.7 m. We used phase-modulation SLMs (Holoeye Photonics AG, ‘PLUTO’) to display holograms. Table 1 shows the computation time of the RS plane method by the CPU and GPU. Here, we arranged three GPUs on the PC to generate holograms. The values in Table 1 represent the total computation time involved in acquiring wavefront information, Fresnel diffraction and hologram calculation. Here the transfer time HtoD is the transfer time from the host PC to the device (i.e. the GPU), while the transfer time DtoH is the transfer time from the device to the host PC. As shown in Table 1, the computation time required by the GPU is approximately 300–500 times faster than that of the CPU, and the frame-rate of hologram generation from 3,072 × 3,072-pixel RS images corresponds to approximately 15 frames per second.

**Table 1:** Computation time and computational time details for hologram generation (CPU computation time is the average of 10 measurements; GPU computation time is the average of 1,000 measurements).

RS image resolution [pixels]	2,048 × 2,048		3,072 × 3,072		4,096 × 4,096	
Processor	CPU	GPU	CPU	GPU	CPU	GPU
Transfer time HtoD [ms]		1.9		3.1		5.7
Wavefront information acquisition [ms]	1,050	1.1	2,910	2.5	4,380	4.4
Propagation calculation [ms]	6,360	8.0	30,180	37.3	31,050	38.1
Hologram calculation [ms]	210	1.9	900	3.8	990	6.7
Transfer time DtoH [ms]		0.2		0.2		0.2
Drawing time [ms]	12.0	12.0	12.0	12.0	12.0	12.0
Total computation time [ms]	7,632	25.1	34,002	58.9	36,432	67.1

**Figure 1:**
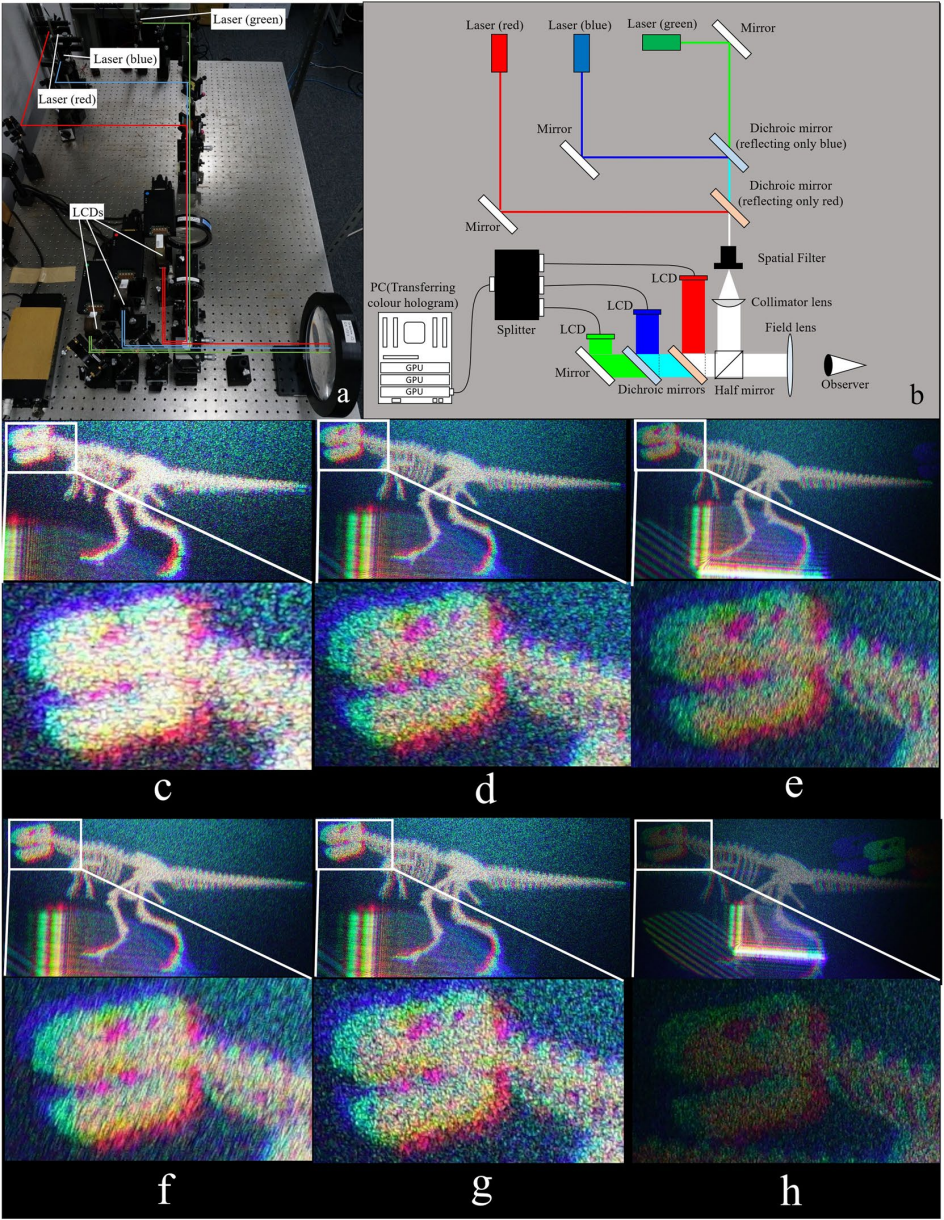
(**a**) Optical system to reconstruct colour 3D images by electro-holography, (**b**) overview of the hologram setup. Image reconstructed from six RS images at resolutions of (**c**) 2,048 × 2,048, (**d**) 3,072 × 3,072, (**e**) 4,096 × 4,096, (**f**) 3,072 × 3,072, (**g**) 3,072 × 3,072 and (**h**) 6,144 × 6,144, and the resolution after expanding by zero padding is (**c**) 4,096 × 4,096, (**d**) 6,144 × 6,144, (**e**) 8,192 × 8,192 pixels, (**f**) 4,096 × 4,096, (**g**) 8,192 × 8,192 and (**h**) 8,192 × 8,192.

**Figure 2:**
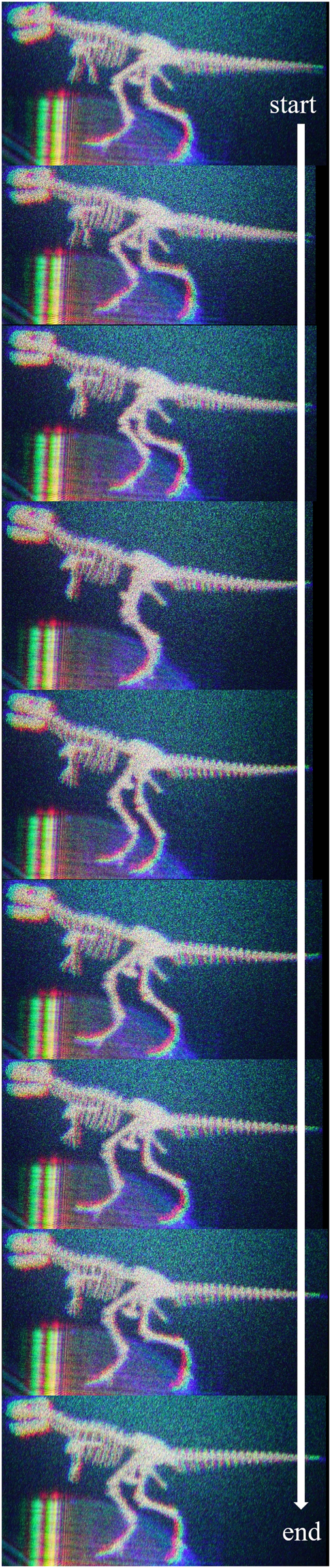
Reconstructed images (several frames in Supplementary Video 1).

Figure 1a shows the optical system to reconstruct colour 3D images by electro-holography^15^. Figure 1b shows a schematic of the electro-holographic reconstruction system in this experiment. Figure 1c–e show images reconstructed from three RS images at resolutions of 2,048 × 2,048, 3,072 × 3,072 and 4,096 × 4,096 pixels with zero padding of 2 *N* × 2 *N* pixels (described in the Methods section). Figure 1f–h show images reconstructed from three RS images at resolutions of 3,072 × 3,072, 3,072 × 3,072 and 6,144 × 6,144 pixels with zero paddings of 4.096 × 4,096, 8,192 × 8,192 and 8,192 × 8,192 pixels, respectively (described in the Discussion section). A comparison of the enlarged view of each reconstructed image reveals that the resolution of the RS image increases when the quality of the reconstructed images improves. This is because reconstructed image quality in the RS plane method improves with an increase in the number of elemental images of the RS image. Because we fixed the resolution of each elemental image at 16 × 16 pixels, the number of elemental images is proportional to the resolution of the RS images. Figure 2 and Supplementary Video 1 shows the real-time reconstructed moving pictures from eighty 3,072 × 3,072 RS images obtained using the optical system. Supplementary Video 2 shows the manner in which the program generates holograms and reconstructs 3D images in real time. Figures 1c–h, 2, Supplementary Videos 1 and 2 demonstrate that the CGH calculation is successfully performed in real time.

**Figure 3:**
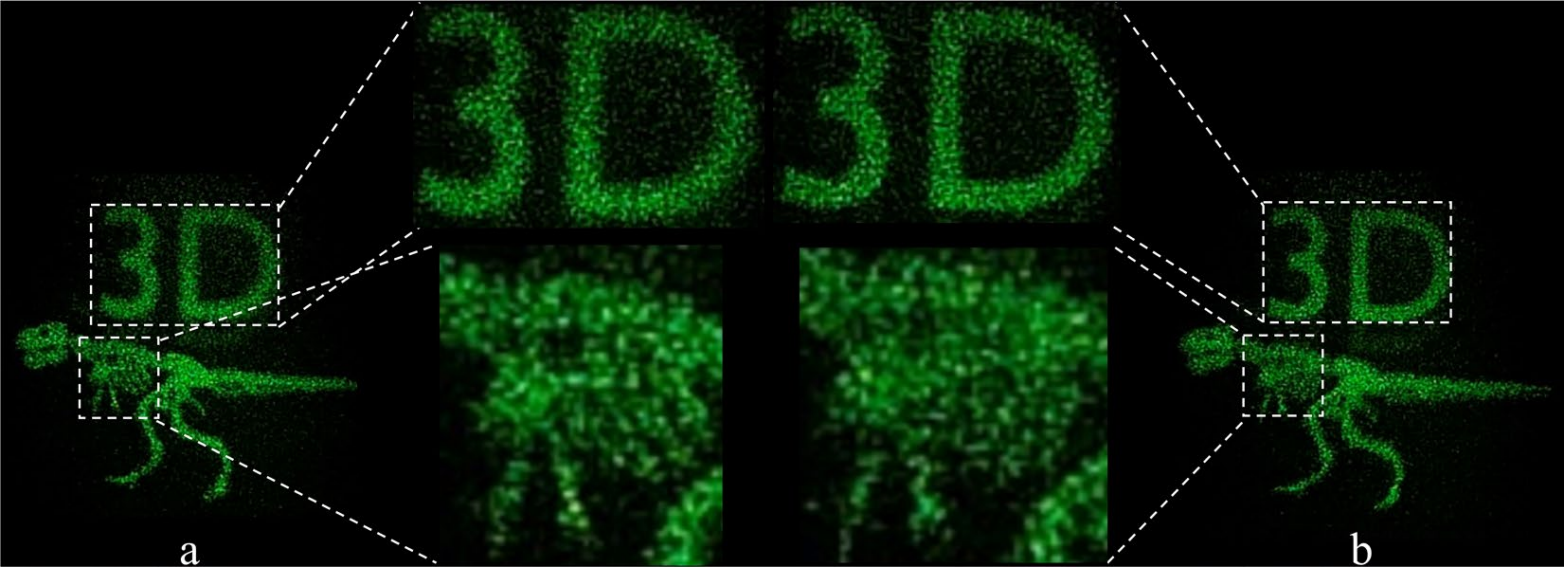
Image reconstructed from an RS image generated from two objects (the dinosaur and the text “3D” ~2 cm behind the dinosaur) at a resolution of 2,048 × 2,048, and the resolution after expanding by zero padding is 4,096 × 4,096. Focusing a digital camera on (**a**) the dinosaur and (**b**) the “3D” text.

Even in the image shown in Fig. 1e, the depth difference and volume effect in the 3D space are not clearly observed. Next, we placed an additional object containing the text “3D” ~2 cm behind the dinosaur and generated an RS image and a hologram from the two objects. We captured two reconstructed images by focusing a digital camera on the dinosaur (Fig. 3a) and the “3D” text (Fig. 3b); the “3D” text and dinosaur images were blurred. These images indicate that the reconstructed holographic images showed a depth difference and volume effect in the 3D space.

## Discussion

The difference in computation time is small between the 3,072 × 3,072 and 4,096 × 4,096 pixel RS images (Table 1). In other words, there is little difference in the computation time of the wavefront propagation, which requires the most time for hologram generation, between 3,072 × 3,072 and 4,096 × 4,096 RS images. This results from the computational efficiency of the two-dimensional (2D) FFTs. A convolution-based Fresnel diffraction calculation was employed to perform wavefront propagation (described in the Methods section); therefore, it takes more time to calculate a 2D FFT and inverse 2D FFT than other calculations. In addition, we employed zero padding to expand the resolution of the RS images from *N* × *N* pixels to 2 *N* × 2 *N* pixels (described in the Methods section). Table 2 shows the FFT computation time with a GPU and image resolution changes by zero padding. The computational efficiency of FFT reaches a maximum value when the number of elements is a power of two because the number of FFT calculations equals O(2*n* log *n*), where *n* is the number of elements. There is little difference in computational time between images with 3,072 × 3,072 pixels and those with 4,096 × 4,096 pixels. Moreover, the computational time at a resolution of 8,192 × 8,192 pixels is shorter than that at 6,144 × 6,144 pixels. Therefore, we consider that the reduction of wavefront propagation computation cost is effective for accelerating hologram generation. Figure 1f and g show the images reconstructed from the RS image at a resolution of 3,072 × 3,072 pixels. The resolution of the RS image was expanded to 4,096 × 4,096 (Fig. 1f) and 8,192 × 8,192 (Fig. 1g) pixels by zero padding. As shown in Table 2, a resolution of 4,096 × 4,096 is appropriate from a computational efficiency perspective. In addition, comparing the enlarged view of each reconstructed image, both Fig. 1f and g have the same image quality as Fig. 1d, which was obtained by expanding the RS image with a resolution of 3,072 × 3,072 pixels to 6,144 × 6,144 pixels. This indicates that the number of pixels required for zero padding is practically less than 2 *N* × 2 *N*, and it is possible to calculate the hologram and reconstruct the desired image from 3,072 × 3,072 expanded to 4,096 × 4,096 pixels using zero padding without practical problems. Supplementary Video 3 shows real-time reconstructed moving pictures from eighty 3,072 × 3,072 RS images expanded to 4,096 × 4,096 pixels. Table 3 shows the hologram generation computation time for expanding the RS image from 3,072 × 3,072 pixels to 4,096 × 4,096 pixels. We achieved approximately 30 frames per second by setting the resolution after conducting zero padding to an efficient resolution for FFT.

**Table 2:** Relationship between image resolution and FFT computation time.

Image resolution [pixels]	FFT computation time by cuFFT [ms]
1,024 × 1,024	0.229
2,048 × 2,048	0.871
3,072 × 3,072	3.292
4,096 × 4,096	3.426
5,120 × 5,120	10.799
6,144 × 6,144	15.734
7,168 × 7,168	21.437
8,192 × 8,192	13.988
9,216 × 9,216	35.229
10,240 × 10,240	43.513

**Table 3:** Computation time and computational time details for hologram generation (GPU computation time is the average of 1,000 measurements).

RS image resolution [pixels]	3,072 × 3,072
Processor	GPU
Transfer time HtoD [ms]	3.1
Wavefront information acquisition [ms]	2.5
Propagation calculation [ms]	8.3
Hologram calculation [ms]	3.8
Transfer time DtoH [ms]	0.2
Drawing time [ms]	12.0
Total computation time [ms]	29.9

In addition, it is impossible to calculate a hologram from a 6,144 × 6,144-pixel RS image by performing 2 *N* × 2 *N* zero padding due to GPU memory buffer overflows that occur in FFT for wavefront propagation when the RS image resolution is expanded to 12,288 × 12,288 pixels. On the other hand, a hologram can be calculated from a 6,144 × 6,144 RS image by setting the zero padding resolution to 8,192 × 8,192 pixels, which reduces the amount of memory used. Figure 1h shows an image reconstructed from a 6,144 × 6,144 RS image. We confirm that the image reconstructed from the 6,144 × 6,144 RS image in Fig. 3c demonstrates higher image quality than the 4,096 × 4,096 RS image in Fig. 1e.

## Methods

### Hologram generation from RS plane

**Figure 4:**
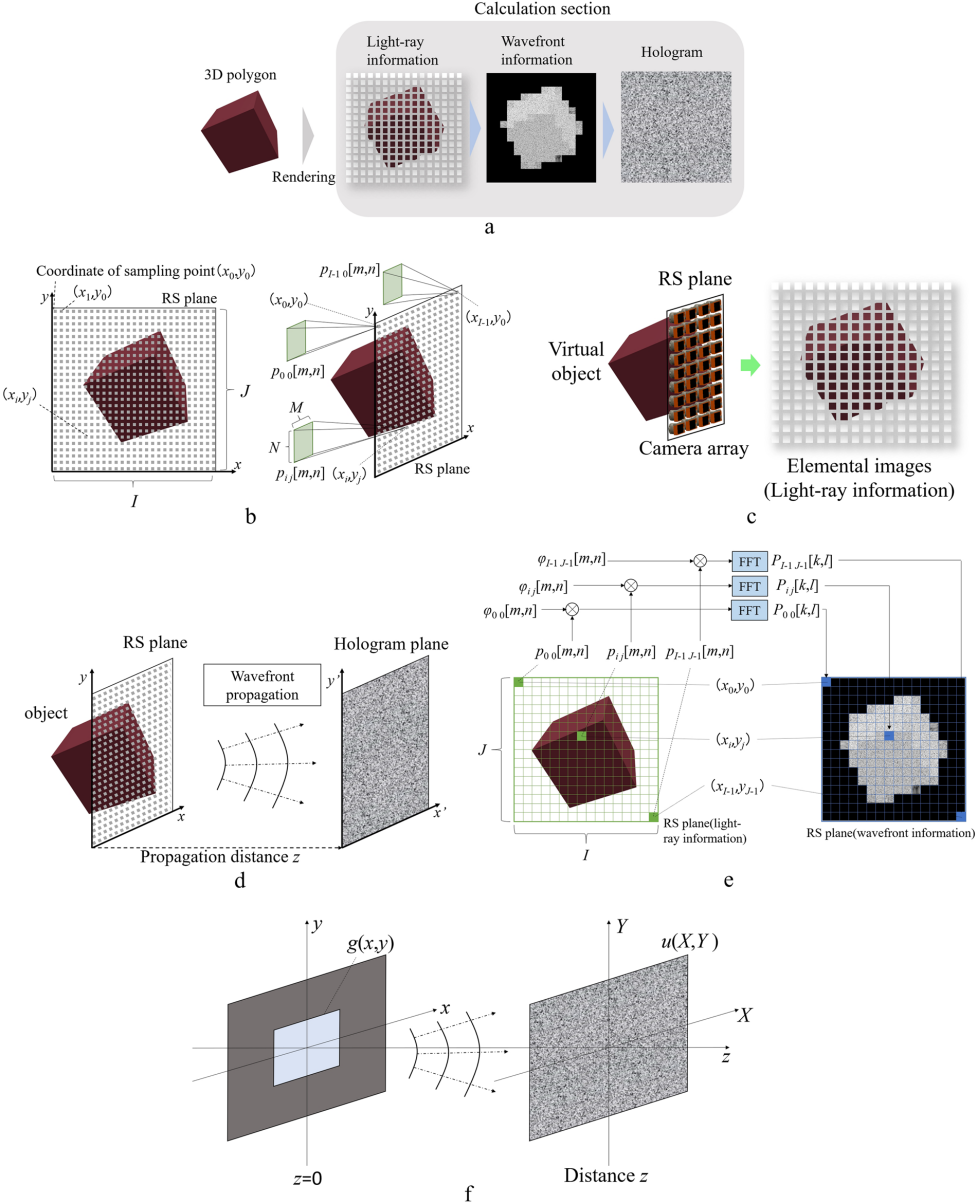
(**a**) Flow of RS plane-based hologram generation, (**b**) Model of RS plane and elemental images at each light-ray point, (**c**) method to acquire elemental images, (**d**) Position of RS plane and hologram plane, (**e**) method to acquire wavefront information and (**f**) Fresnel diffraction schematic.

This section discusses the hologram generation method based on the RS plane^24^. Figure 4a shows the flow of the method. Here, a method that involves placing an RS plane near an object and placing a hologram plane distant from the object is used^24^. As shown in Fig. 4b, in this method, the first elemental images *p*_*i j*_[*m*, *n*] are sampled from the object at the (*x*_*i*_, *y*_*j*_) coordinates on the RS plane. Here, *m* and *n* denote the x- and y-coordinates in each elemental image comprising *M* × *N* pixels, respectively, and *I* and *J* denote the number of elemental images in the horizontal and vertical directions, respectively. As shown in Fig. 4c, the elemental images are considered 2D images with different viewpoint positions of the object as obtained by a camera array. As a result, each pixel of the elemental image maintains information related to different light-rays with different intensities and directions; thus, it is possible to represent the light-ray using plane waves, i.e. the angular spectra^24^. However, the number of elemental images is equivalent to the resolution of the reconstructed image, and the resolution of the elemental image is equivalent to the angular resolution of the reconstructed image. Therefore, when the resolution of the elemental image is fixed, the resolution of the reconstructed image increases depending on the resolution of the RS image.

Figure 4d shows the relationship between the positions of the RS and hologram planes. The previous section discussed the acquisition of angular spectra by sampling light-ray information from the object. However, as shown in Fig. 4b, the RS plane is separate from the hologram plane; therefore, it is necessary to calculate the wavefront propagation from the RS plane to the hologram plane^24^. This section explains the method used to transform angular spectra to wavefront propagation. Here, the angular spectra acquired in the previous section are denoted by *A*(*f*_*X*_, *f*_*Y*_, 0). Subsequently, the complex optical field *U*(*x*, *y*, 0) required for wavefront information is expressed as follows:

1 $$U(x,\,y,\,0)=\int {\int }_{-\infty }^{\infty }A({f}_{X},{f}_{Y},0)\exp [-2\pi ({f}_{X}x+{f}_{Y}y)]d{f}_{X}d{f}_{Y}$$

here *f*_*X*_ and *f*_*Y*_ denote spatial frequencies in *x* and *y* directions. The FFT is expressed as follows:

2 $$G({f}_{x},\,{f}_{y},)={\rm{FFT}}\{g({f}_{x},{f}_{y})\}={\int }_{-\infty }^{\infty }g({x}_{0},{y}_{0})\exp \,[-2\pi ({f}_{x}{x}_{0}+{f}_{y}{y}_{0})]d{x}_{0}d{y}_{0}$$

furthermore, by comparing Eqs. (1) and (2), it can be seen that *U*(*x*, *y*, 0) is equivalent to the Fourier-transformed *A*(*f*_*X*_, *f*_*Y*_, 0). Therefore, *U* (*x*, *y*, 0) can be expressed as follows:

3 $$U(x,\,y,\,0)={\rm{FFT}}\{A({f}_{X},{f}_{Y},0)\}.$$

**Figure 5:**
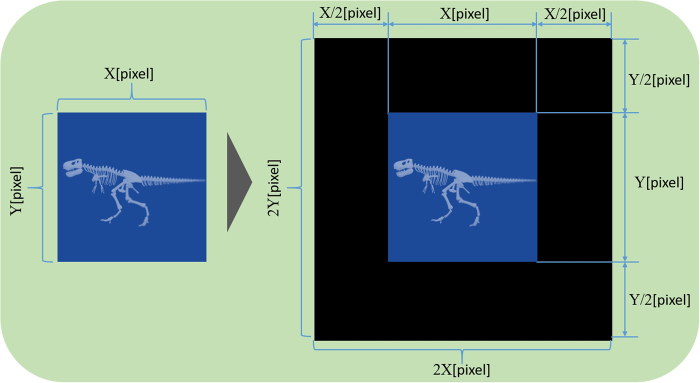
Schematic of zero padding.

Figure 4e summarises the procedure used to transform light-ray information to wavefront information. The left side of the figure shows the elemental images maintaining light-ray information on the RS plane, and the right side represents the transformed light-ray information (i.e. the wavefront information). Here, the elemental image *p*_*i j*_[*m*, *n*] at (*x*_*i*,_ *y*_*j*_) was added by a random phase *φ*_*i j*_[*m*, *n*] to diffuse light. The range of the random phase corresponds to (0, 2π). Subsequently, the wavefront information *P*_*i j*_[*k*, *l*] is obtained via FFT as follows:

4 $${P}_{ij}[k,l]={\rm{F}}{\rm{F}}{\rm{T}}\{{p}_{ij}[m,n]\exp \,(j{\phi }_{ij}[m,n])\}$$

here j denotes the imaginary unit.  Subsequently, wavefront information (i.e. the complex optical field) on the hologram plane is calculated by a propagation calculation from the RS plane to the hologram plane. Here, a convolution-based Fresnel diffraction calculation is used. An aperture plane (i.e. the complex optical field *g*(*x*, *y*)) and a screen *u*(*X*, *Y*) are assumed as shown in Fig. 4f, and the complex optical field *u*(*X*, *Y*) is represented by Fresnel diffraction and expressed as follows:

5 $$u(X,Y)=\frac{1}{j\lambda z}\exp \,(-jkz)\int {\int }_{-\infty }^{\infty }g(x,y)\exp \,\{-jk\frac{{(X-x)}^{2}+{(Y-y)}^{2}}{2z}\}dxdy$$

here, *k* = 2π/λ denotes the wave number and λ denotes the wavelength. Note that Eq. (5) is a convolution integral and can be represented using FFT as follows:

6 $$\begin{array}{c}u(X,Y)={{\rm{FFT}}}^{-1}\{{\rm{FFT}}\{g(x,y)\}\cdot {\rm{FFT}}\{h(x,y)\}\},\\ h(x,y)=\frac{1}{j\lambda z}\exp \,\{-\frac{jk}{2z}({(X-x)}^{2}+{(Y-y)}^{2}+2{z}^{2})\}\end{array}$$

here, Fresnel diffraction is used, and the complex optical field *W*_*H*_[*k*_*H*_, *l*_*H*_] on the hologram from the wavefront information *W*_*RS*_[*k*_*RS*_, *l*_*RS*_] on the RS plane is expressed as follows:

7 $${W}_{H}[{k}_{H},{l}_{H}]={{\rm{FFT}}}^{-1}\{{\rm{FFT}}\{{W}_{RS}[{k}_{RS},{l}_{RS}]\}\cdot {\rm{FFT}}\{h(x,y)\}\}$$

where *k*_*H*_ is 0, 1, …, *IM* − 1 and *l*_*H*_ is 0, 1, …, *JN* − 1. In Eq. (7), the propagation calculation is represented by only a 2D FFT. Subsequently, zero padding is performed to prevent any aliasing noise from overlapping the desired reconstructed image. Here, we expand the resolution of RS images of *N* × *N* pixels to 2 *N* × 2 *N* pixels by employing zero padding, as shown in Fig. 5.

The complex optical field on the hologram is then converted to a phase-only hologram because a phase-modulated SLM was used. Therefore, a kinoform-type phase hologram *H*[*k*_*H*_, *l*_*H*_] can be calculated as follows:

8 $$H[{k}_{H},{l}_{H}]={\rm{\arg }}\,({W}_{H})={\tan }^{-1}\frac{{\rm{Im}}({W}_{H})}{{\rm{Re}}({W}_{H})}.$$

### 3D model specifications

**Figure 6:**
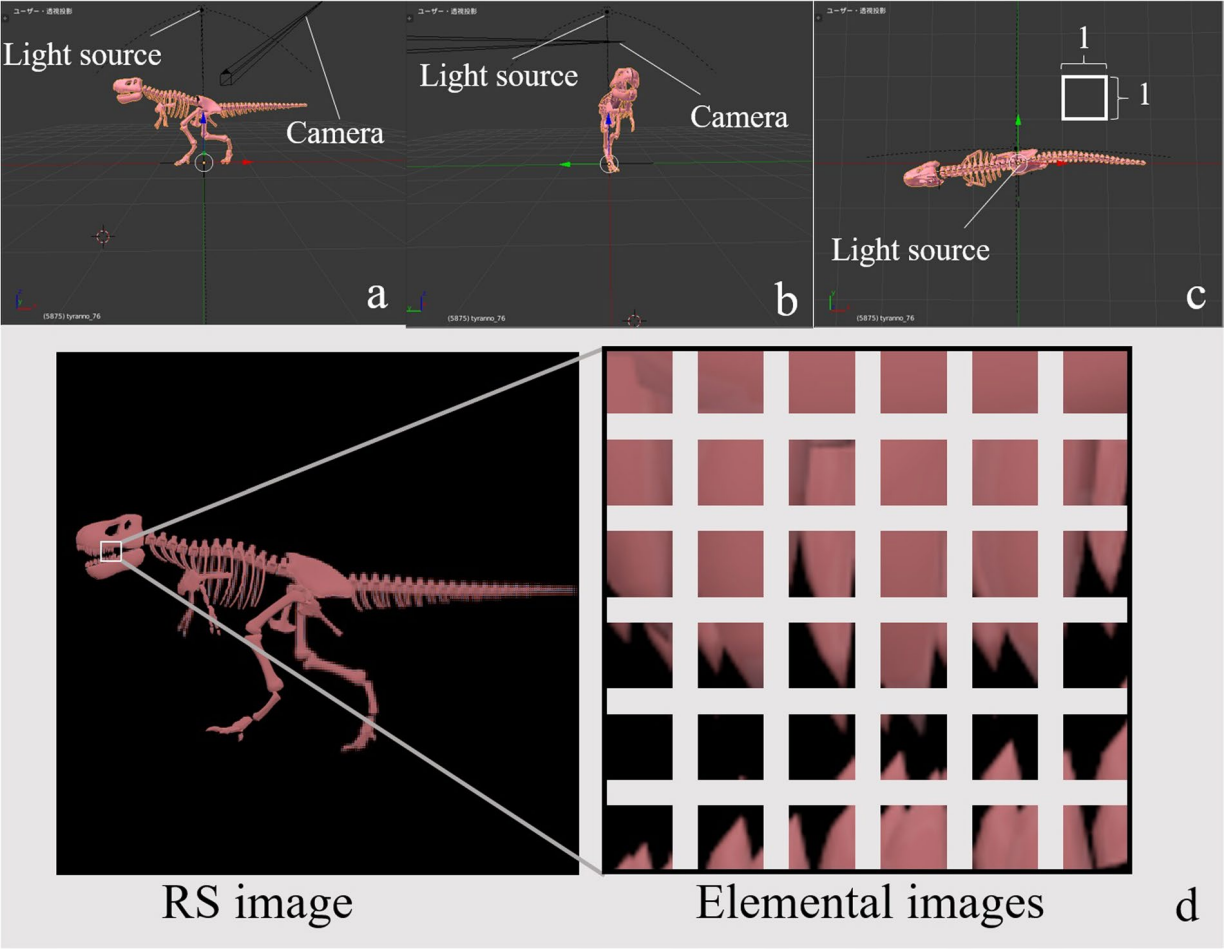
Example virtual 3D object in Blender from (**a**) front, (**b**) side and (**c**) top views. (**d**) Example RS and elemental images.

Elemental images were acquired by scanning with a virtual camera using the Blender 3DCG modelling tool^25^. Here, the 3DCG object data are created by us. Figure 6 shows the front, side and top views of the virtual objects.

Note that the unit length is defined as 1 in Blender. A unit length of 1 in real space depends on the pixel pitch *p*, camera scanning distance *d* and the resolution of RS image *s*, and is obtained as follows:

9 $$({\rm{The}}\,{\rm{length}}\,{\rm{of}}\,{\rm{1}})=\frac{(p\times s)}{d}.$$

We scanned the virtual camera for 4.0 on the x- and y-axis when the elemental images were acquired. Figure 6d shows the elemental images. Here, the resolutions of the RS images are 2,048 × 2,048, 3,072 × 3,072 and 4,096 × 4,096 pixels, and the number of elemental images for each RS image is 128 × 128 ply, 192 × 192 ply and 256 × 256 ply. The resolution for all elemental images is 16 × 16 pixels, which is the same for all RS images.

### Implementing GPU method

This section explains the manner in which the hologram generation method based on the RS plane was implemented. The implementation was executed using three GPUs, and red, green and blue holograms were calculated by each GPU. Then, we used one of three GPUs for delivering a hologram to the SLMs. We transferred the colour holograms to the RGB splitter, which divided the colour-synthesized input signal into three output signals (red, green and blue) via digital visual interface cables, and monochromatic holograms were displayed on the SLMs.

Note that acquiring wavefront information from light-ray information requires a similar number of FFTs as the number of elemental images. The process of 2D FFT for each elemental image corresponds to one of the heaviest computational processes in this method. This is followed by using the cuFFT library, an FFT CUDA library. Moreover, cufftPlanMany^26^, which is one of the function of cuFFT library, is used to accelerate the 2D FFT process of elemental images as cufftPlanMany can parallelise several 2D FFTs and can realise speedy execution of the 2D FFT process.

Note that the calculations in Eq. (8) are independent relative to each pixel. The calculation is parallelised by allotting the calculation of each pixel to each GPU thread. The numbers of blocks and threads of a GPU correspond to those of the elemental images and pixels of each elemental image, respectively.

## Electronic supplementary material

Supplementary Video 1

Supplementary Video 2

Supplementary Video 3
